# Transcriptome Profiling Analysis of the Testis After Eyestalk Ablation for Selection of the Candidate Genes Involved in the Male Sexual Development in *Macrobrachium nipponense*

**DOI:** 10.3389/fgene.2021.675928

**Published:** 2021-05-31

**Authors:** Shubo Jin, Yin Fu, Yuning Hu, Hongtuo Fu, Sufei Jiang, Yiwei Xiong, Hui Qiao, Wenyi Zhang, Yongsheng Gong, Yan Wu

**Affiliations:** ^1^Key Laboratory of Freshwater Fisheries and Germplasm Resources Utilization, Ministry of Agriculture, Freshwater Fisheries Research Center, Chinese Academy of Fishery Sciences, Wuxi, China; ^2^Key Laboratory of Marine and Estuarine Fisheries, Ministry of Agriculture, East China Sea Fisheries Research Institute, Chinese Academy of Fishery Sciences, Shanghai, China; ^3^Wuxi Fisheries College, Nanjing Agricultural University, Wuxi, China

**Keywords:** *Macrobrachium nipponense*, eyestalk ablation, testis, male sexual development, NF_k_Bα

## Abstract

The eyestalk of crustacean species secretes many hormones, affecting the process of reproduction, molting, metabolism of glucose, and other functions in crustaceans. In this study, important metabolic pathways and candidate genes involved in the male sexual development were identified through performing the transcriptome profiling analysis of the testis after the ablation of eyestalk from *Macrobrachium nipponense*. The histological observations revealed that the testis development became vigorous after eyestalk ablation, indicating that the hormones secreted by the eyestalk have negative effects on the testis development in *M. nipponense*. Transcriptome profiling analysis revealed that 1,039, 1,226, and 3,682 differentially expressed genes (DEGs) were identified between normal prawns (CG) vs single-side eyestalk ablation prawns (SS), SS vs double-side eyestalk ablation prawns (DS), and CG vs DS, respectively, indicating that the ablation of double-side eyestalk has more significant regulatory roles on male sexual development than that of single-side ablation, which was consistent with the histological observations. Lysosome, Apoptosis, Glycolysis/Gluconeogenesis, and Insulin signaling pathway were the main enriched metabolic pathways in all of these three comparisons, and the important genes from these metabolic pathways were also selected. The qPCR verifications of 10 DEGs from these metabolic pathways were the same as those of RNA-seq. The qPCR, *in situ* hybridization, and RNA interference analysis of Mn-NF_k_Bα revealed that NF_k_Bα has a positive regulatory effect on testis development. This study provided new insights on male sexual development in *M*. *nipponense*, promoting the studies on male sexual development in other crustaceans as well.

## Introduction

The oriental river prawn, *Macrobrachium nipponense* (Crustacea; Decapoda; and Palaemonidae), is widely distributed in China and other Asian countries (Cai and Shokita, 2006; Salman et al., 2006; Ma et al., 2011), which is an important commercial species with annual aquaculture production that reached 205,010 tons in 2016 (Wang et al., 2019). The same as other *Macrobrachium* species, male prawns grow faster and reach larger size at the harvest time (Ma et al., 2011). Thus, male prawns are preferred in the *M*. *nipponense* aquaculture. But the rapid development of the testis in the reproductive season is another main problem that restricted the sustainable development of *M*. *nipponense*. Previous studies revealed that the testis of a newborn *M*. *nipponense* can reach sexual maturity within 40 days after hatching (Jin et al., 2016). Thus, inbreeding will happen between the newborn prawns. Inbreeding will lead to the decrease of the ability of resistance to adversity in their offspring, the small scale of market prawn, and the degradation of germplasm resources. Therefore, it is urgently needed to fully understand the male sexual determination and development mechanism, especially for the identification of the key metabolic pathways and genes involved in the mechanism of male sexual determination and development in *M*. *nipponense*, with the aims of establishing the technique to produce all male progeny on a commercial scale and regulating the process of testis development.

The eyestalk of crustacean species has many neurosecretory structures. The X-organ–SG complex (XO–SG) is located in the eyestalk in crustaceans, which was identified as a principal neuroendocrine gland (Hopkins, 2012). It stores and releases the crustacean hyperglycemic hormone (CHH) superfamily neurohormones, including CHH, gonad-inhibiting hormone (GIH), molt-inhibiting hormone (MIH), ion transport peptides, and mandibular organ-inhibiting hormone (MOIH), playing essential roles in reproduction (Treerattrakool et al., 2011, 2013; Revathi et al., 2013), molting (Pamuru et al., 2012; Salma et al., 2012; Shen et al., 2013), metabolism of glucose (Santos et al., 1997; Almeida et al., 2004), and other functions (Tiu and Chan, 2007; Sainz-Hernández et al., 2008; Diarte-Plata et al., 2012). Knockdown of the expression of GIH by RNA interference (RNAi) promotes the ovarian development in *M*. *nipponense* (Qiao et al., 2015). Knockdown of the expression of MIH by RNAi promotes the molting in *M*. *nipponense* (Qiao et al., 2018). CHH has been proven to promote testis development in *M*. *nipponense* (Jin et al., 2013b). Many previous studies have proven that the ablation of eyestalk from crustacean species has positive effects on the expression of insulin-like androgenic hormone (IAG; Sroyraya et al., 2010; Chung et al., 2011; Guo et al., 2019). IAG is an important gene playing essential roles in the male sexual differentiation and development in many crustacean species (Ventura et al., 2009, 2011). Knockdown of the expression of IAG resulted in sex reversal in *Macrobrachium rosenbergii*, and the all-male progeny were produced when the “reversal females” were mating with normal male *M. rosenbergii* (Ventura et al., 2012).

It is widely acknowledged that the testis plays essential regulatory roles in reproduction, sexual maturity, and sex differentiation. Previous male reproductive studies have been conducted at the molecular and cellular levels in *M*. *nipponense* (Qiu et al., 1995; Yang et al., 1999; Cao, 2006; Guo, 2007). A total of 52 candidate male reproduction-related genes were identified from the testis cDNA library of *M*. *nipponense* (Qiao et al., 2012). An integrated analysis of metabolomes and transcriptomes was also performed in the testis between the reproductive season and non-reproductive season, in order to select candidate male reproduction-related metabolites and genes, regulated by different water temperature and illumination time (Jin et al., 2020). Some candidate genes from these constructed testis transcriptomes have proven their functions in the mechanism of male sexual differentiation and development (Zhang et al., 2013a,b,c). These studies dramatically improved the studies on the mechanism of male sexual differentiation and development in *M*. *nipponense*. However, the effects of eyestalk on male sexual differentiation and development were still unclear.

In this study, we aimed to select the vital metabolic pathways and genes involved in the male sexual differentiation and development in *M*. *nipponense* through performing the transcriptome profiling analysis of the testis after the ablation of single-side and double-side eyestalks. The functions of nuclear factor kappa B inhibitor alpha (NF_k_Bα) were further analyzed in-depth by using qPCR analysis, *in situ* hybridization, and RNAi. This study provided valuable evidences on the studies of male sexual differentiation and development in *M*. *nipponense*, as well as other crustacean species.

## Materials and Methods

### Ethics Statement

We obtained the permission from the committee of Freshwater Fisheries Research Center and the Tai Lake Fishery Management Council during the experimental programs. MS222 anesthesia was used to sedate the prawns and shear the tissues.

### Sample Collection

A total of 600 healthy male *M. nipponense* prawns were collected from a wild population in Tai Lake, Wuxi, China (120°13′44″E, 31°28′ 22′N) with body weights of 3.34–4.76 g. All the samples were randomly divided and transferred to three 500-L tanks and maintained in aerated freshwater for 3 days with dissolved oxygen of ≥6 mg/L prior to the tissue collection. The three groups were normal prawns (CG), single-side eyestalk ablation prawns (SS), and double-side eyestalk ablation prawns (DS). The death rate of DS prawns was 13.7 and 8.6% higher than that of CG prawns and SS prawns, respectively. The testis was collected from these three groups after 7 days of treatment and immediately preserved in liquid nitrogen until use for transcriptomic analysis.

### Transcriptomic Profiling Analysis

The comparative transcriptome analysis of androgenic gland between the CG, SS, and DS was performed. In order to ensure the sufficient amount of RNA samples, the testis from at least 30 prawns were pooled to form one biological replicate, and three biological replicates were sequenced for all of these three groups. Thus, a total of nine libraries were generated for sequencing. The experimental process of transcriptome sequencing has been well described in the previously published studies (Jin et al., 2013a, 2017, 2020). Briefly, the total RNA from each pooled sample was extracted by using RNAiso Plus Reagent (TaKaRa), following the manufacturer’s instructions. The concentration of total RNA was measure by a spectrophotometer (Eppendorf), and the integrity was measured by using a 2100 Bioanalyzer (Agilent Technologies, Inc.) with a minimum RNA integrity number (RIN) value of 7.0. A total of 4 μg of total RNA was used to construct the library, and Illumina HiSeq 2500 sequencing platform was used to perform the sequencing under the parameter of PE150.

Raw data of fastq format were firstly processed using Trimmomatic with default parameters (Bolger et al., 2014). The clean reads were assembled into expressed sequence tag clusters (contigs) and *de novo* assembled into transcripts by Trinity (version 2.4) with paired-end method with default parameters after removing the adaptor and low-quality sequences (Grabherr et al., 2011). The gene annotation was then performed in the NR protein, prior to Gene Ontology (GO), the Clusters of Orthologous Groups of proteins (COG), and the Kyoto Encyclopedia of Genes and Genomes (KEGG) analyses, using an *E*-value cutoff of 10^–5^ (Jin et al., 2013a). GO (Ashburner et al., 2000), COG (Tatusov et al., 2003), and KEGG (Minoru et al., 2008) analyses were annotated by using Blast2go software and Blast software. The criteria of false discovery rate < 0.05 was used to filter the differentially expressed genes (DEGs) by EB-seq algorithm (Benjamini et al., 2001).

### qPCR Analysis

A total of 15 male and female prawns were collected from a wild population in Tai Lake with body weights of 3.12–3.87 and 1.97–2.54 g, respectively, in order to obtain tissues for qPCR analysis in different mature tissues. All the samples were transferred to 500-L tanks and maintained in aerated freshwater for 3 days with dissolved oxygen of ≥6 mg/L prior to the tissue collection. Different mature tissues included the testis, ovary, hepatopancreas, muscle, eyestalk, gill, heart, and brain (*N* = 5). Specimens for the different stages of post-larval developmental stages were from the full-sibs population, collected during their maturation process (*N* = 5).

qPCR was performed on the Bio-Rad iCycler iQ5 Real-Time PCR System (Bio-Rad), which was used to carry out the SYBR Green RT-qPCR assay. The procedure has been well described in detail in previous studies (Zhang et al., 2013a,b,c). Briefly, the total RNA from each tissue was extracted by using RNAiso Plus Reagent (TaKaRa), following the manufacturer’s instructions. The concentration of total RNA was measured by a spectrophotometer (Eppendorf), and the integrity was measured by agarose gel. Approximately 1 μg of total RNA from each tissue was used for first-strand cDNA synthesis by using iScript^TM^ cDNA Synthesis Kit Perfect Real Time (BIO-RAD), following the manufacturer’s instructions. Amplifications were performed in a 96-well plate with a 25-μl reaction volume containing 12.5 μl of 2 × Ultra SYBR Mix (CWBIO), 0.5 μl of each primer, 1 μl of cDNA template, and 10.5 μl of PCR-grade water. The thermal profile for qPCR was 95°C for 10 min, followed by 40 cycles of 95°C for 15 s and 60°C for 1 min. Each tissue was performed in triplicate. The relative gene expression was calculated based on the 2^–Δ^ ^Δ^ ^CT^ comparative CT method (Livak and Schmittgen, 2001). The primers used for qPCR verification of important DEGs are listed in Table 1. The primers used for qPCR analysis of Mn-NF_k_Bα are listed in Table 2. EIF was used as the reference gene in this study (Hu et al., 2018). Different concentrations of testis cDNA templates were used to measure the amplification efficiency of Mn-NF_k_Bα and EIF, including undiluted, two times diluted, four times diluted, and eight times diluted samples. The slope of the Mn-NF_k_Bα and EIF at different concentrations of diluted samples was 1.412 and 1.423, respectively, indicating that the amplification efficiency between the Mn-NF_k_Bα and EIF is the same in this study.

**TABLE 1:** Primers used for qPCR verification.

Primer	Sequence
Sialin-F	ATCAAAGGAATGTCTGCTACCGT
Sialin-R	TCAGGTAAATCGTTCCAGGGATG
Alpha-F	CAACGACTTTGTCACCAGGAAAA
Alpha-R	TGGTATTCCCTGACCCCATCTAT
ASK1-F	GAATTCTCTCGGAGCATATCCGT
ASK1-R	TCTTCAGGAGGTAGAACCCATCT
NF_k_Bα-F	CATGGTGACCCAGTTAACCAGA
NF_k_Bα-R	CGTCAAGTGTTGCAGGATTTCTT
TGF-F	CATCTTTACCAGAGTGTGTGGGA
TGF-R	CTGCTTACGAATACCCTGTTCCT
ADP-F	CACACCGATGTTTACTTCTGGGA
ADP-R	CAACACATGATCTCCTGGCTGAA
PPT-F	GCACTTGGAAGACGAGATGATTG
PPT-R	GGAACCGTGAGTTTGTAGCTTTC
Hexokinase-F	CACAGGATGCTTCTTTGGAGGA
Hexokinase-R	GAGAGTCTTCCCCTGAATCAAGA
Alcohol-F	TAAAACACCATCCCCCAGAGAAG
Alcohol-R	AGGGTAAGTTTGGATCCCTCAAC
Acetyl-F	CCAAGCTTCAGAACGGATACAAC
Acetyl-R	GACGAAACCACCATTCAAAGAGG

**TABLE 2:** Primers used for NFkB analysis.

Primer name	Nucleotide sequence (5′→3′)	Purpose
NF_k_Bα-RTF	CATGGTGACCCAGTTAACCAGA	FWD primer for NF_k_Bα expression
NF_k_Bα-RTR	CGTCAAGTGTTGCAGGATTTCTT	RVS primer for NF_k_Bα expression
EIF-F	CATGGATGTACCTGTGGTGAAAC	FWD primer for EIF expression
EIF-R	CTGTCAGCAGAAGGTCCTCATTA	RVS primer for EIF expression
NF_k_Bα anti-sense Probe	TTCGCGAAAGAGGGAGAGGAGGAATCAGCGATCCCT	Probe for NF_k_Bα ISH analysis
NF_k_Bα sense Probe	AGGGATCGCTGATTCCTCCTCTCCCTCTTTCGCGAA	Probe for NF_k_Bα ISH analysis
NF_k_Bα RNAi-F	TAATACGACTCACTATAGGGGAAAGAGGAAGGCTCCGTTA	FWD primer for RNAi analysis
NF_k_Bα RNAi-R	TAATACGACTCACTATAGGGGGTCCTTCCGGTATGATTTG	RVS primer for RNAi analysis

### *In situ* Hybridization

The mRNA locations of Mn-NF_k_Bα in the testis, androgenic gland, and different reproductive cycles of ovary were analyzed by using *in situ* hybridization. The different reproductive cycles of ovary were collected, according to the previous study (Qiao et al., 2015). The testis and androgenic gland were collected in reproductive season. Primer5 software was used to design the anti-sense and sense probes of chromogenic *in situ* hybridization study and synthesized with DIG signal by Shanghai Sangon Biotech Company. The sequences of anti-sense and sense probes are listed in Table 1. The previous studies have described well the detailed procedures (Jin et al., 2018; Li et al., 2018). Slides were examined under a light microscope for evaluation.

### RNA Interference Analysis

RNA interference was performed to analyze the regulatory roles on Mn-NF_k_Bα in *M*. *nipponense*. The specific RNAi primer with T7 promoter site was designed by using Snap Dragon tools^1^ and is shown in Table 1. The Transcript Aid^TM^ T7 High Yield Transcription kit (Fermentas Inc., United States) was used to synthesize the Mn-NF_k_Bα dsRNA, followed by the procedures of the manufacturer. A total of 300 healthy mature male *M*. *nipponense* were collected with body weight of 3.17–4.96 g and divided into two groups. As described in previous studies (Jiang et al., 2014; Jin et al., 2018), the prawns from experimental group were injected with 4 μg/g of Mn-NF_k_Bα dsRNA, while the prawns from the control group were injected with an equal volume of green fluorescent protein. The NF_k_Bα mRNA expression was investigated in the androgenic gland by qPCR after the injection at 1, 7, and 14 days in order to detect the interference efficiency (*N* ≥ 5). The mRNA expressions of Mn-IAG were also measured in the androgenic gland templates from the same prawns in order to analyze the regulatory relationship between Mn-NF_k_Bα and Mn-IAG.

### Histological Observation

The morphological changes of the testis between different days after RNAi treatment were observed by hematoxylin and eosin (H&E) staining. Five testicular samples were collected after 1, 7, and 14 days of RNAi treatment for H&E staining. The procedures have been described well in previous studies (ShangGuan et al., 1991; Ma et al., 2006). Olympus SZX16 microscope was used to observe the slides (Olympus Corporation, Tokyo, Japan). The various cell types were labeled based on morphological analysis (Jin et al., 2016).

### Statistical Analysis

SPSS Statistics 23.0 was used to measure the statistical differences, estimated by one-way ANOVA followed by least significant difference and Duncan’s multiple range test. Quantitative data were expressed as mean ± SD. *p* < 0.05 indicates a significant difference.

## Results

### Histological Observations of the Testis After Eyestalk Ablation

**FIGURE 1:**
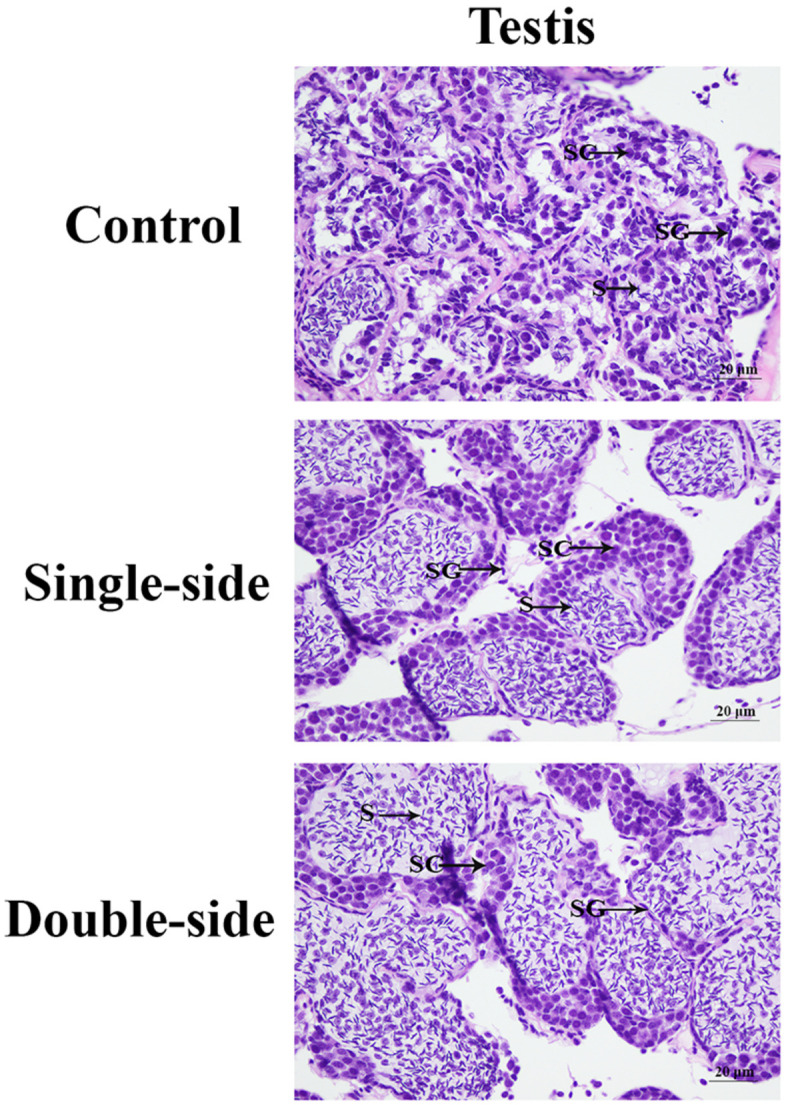
The morphological differences of the testis after the ablation of eyestalk. SG, spermatogonia; SC, spermatocytes; S, sperms; and CT, collected tissue. Scale bars = 20 μm.

Histological observations were performed in order to analyze the morphological changes of the testis after the ablation of eyestalk in *M. nipponense* (Figure 1). The histological observations revealed that the greatest number of spermatogonia was observed in the CG prawns, followed by SS prawns and DS prawns. However, the dominant cells in the DS prawns were sperms, which were more than those in SS prawns and CG prawns. Spermatogonia were rarely observed in the DS prawns.

### Transcriptome Analysis

The transcriptome generated 54,341 non-redundant transcripts with an average length of 1,311.61 bp. The non-redundant transcripts length ranged from 301 to 28,887 bp. The majority of the transcripts was 301–400 bp (23.62%) in length, followed by >2,000 bp (19.61%) and 401–500 bp (13.36%). The complete and duplicated BUSCOs of this assembled transcriptome reached 97.5%, indicating the completeness of this assembled transcriptome.

All of the assembled unigenes were firstly annotated in the Nr (non-redundant) database. A total of 17,660 (32.50%) unigenes were annotated in the Nr database, while the other unannotated unigenes represent novel genes, but the functions need further investigations.

**FIGURE 2:**
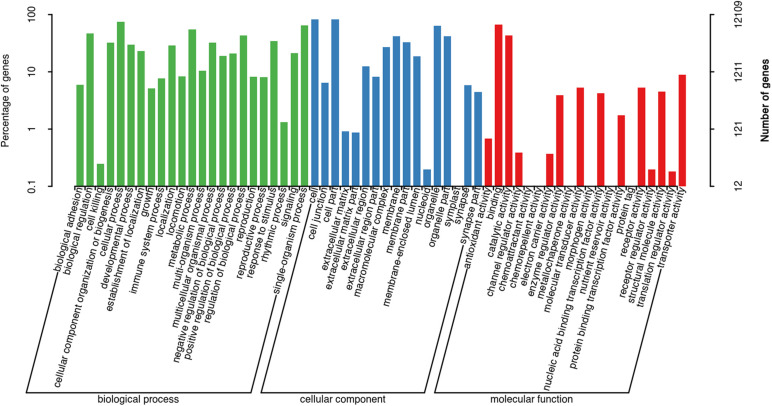
Gene ontology classification of non-redundant transcripts.

**FIGURE 3:**
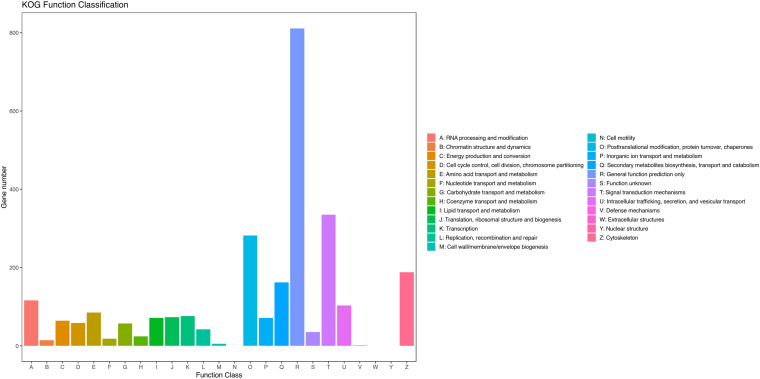
Clusters of orthologous groups of proteins (COG) classification of putative proteins.

The assembled unigenes were then annotated in the GO, COG, and KEGG databases. GO and COG analyses provide a structured vocabulary to describe the transcripts. A total of 12,109 unigenes matched the known proteins in GO database, composed of 60 functional groups (Figure 2). The number of unigenes in each functional group ranged from 1 to 10,057. Cell, Cell part, Cellular process, and Binding represent the main functional groups, in which the number of unigenes was >8,000. A total of 2,509 unigenes were assigned to the matched proteins in COG database, including 22 functional categories (Figure 3). The unigenes in each functional category ranged from 1 to 811. The main functional category includes General function prediction only, Signal transduction mechanisms, and Posttranslational modification, protein turnover, and chaperones, in which the number of unigenes was more than 200.

Kyoto Encyclopedia of Genes and Genomes analysis plays essential roles in releasing the regulatory relationship between the unigenes, assembled in this transcriptome. A total of 4,971 unigenes were matched with the known proteins in the KEGG database involved in the 338 metabolic pathways. The metabolic pathways, in which the number of unigenes was more than 200, included Alzheimer disease, Pathways in cancer, and Huntington disease.

### Identification of Differentially Expressed Genes

The DEGs were identified using the criterion of >2.0 as up-regulatory genes and <0.5 as down-regulatory genes, and *p*-value < 0.05. A total of 1,039 DEGs were identified between CG and SS, including 617 up-regulated genes and 422 down-regulated genes. Eighty-seven metabolic pathways were identified, and the number of DEGs in each metabolic pathway ranged from 1 to 4. A total of 1,226 DEGs were identified between SS and DS, including 739 up-regulated genes and 487 down-regulated genes. A total of 196 metabolic pathways were identified, and the number of DEGs in each metabolic pathway ranged from 1 to 8. A total of 3,682 DEGs were found between CG and DS, including 1,978 up-regulatory genes and 1,704 down-regulatory genes. A total of 285 metabolic pathways were identified, and the number of DEGs in each metabolic pathway ranged from 1 to 56. KEGG analysis revealed that Lysosome, Apoptosis, Insulin signaling pathway, and Glycolysis/Gluconeogenesis were the main enriched metabolic pathways in all of these three comparisons.

Ten important DEGs were identified from these metabolic pathways, which were differentially expressed in at least two comparisons (Table 3). Sialin-like, alpha-L-fucosidase, and acetyl-CoA carboxylase (ACC) were selected from the metabolic pathway of Lysosome. Apoptosis signal-regulating kinase 1 (ASK1), NF_k_Bα, and TGF-beta-activated kinase 1 (TGF) were selected from the metabolic pathway of Apoptosis. Alcohol dehydrogenase class-P (ADP), Palmitoyl-protein thioesterase 1 (PPT1), and Hexokinase (HXK) were selected from the metabolic pathway of Glycolysis/Gluconeogenesis.

**TABLE 3:** Important DEGs through transcriptome profiling analysis.

Name	Accession number	*p* value	CG vs SS	CG vs DS	SS vs DS	Metabolic pathways
				Fold change		
Sialin-like	XP_018006493.1	0.0367	0.32	0.39		Lysosome
Alpha-L-fucosidase	KFM70007.1	0.0105	2.01	2.23		Lysosome; other glycan degradation
Apoptosis signal-regulating kinase 1 (ASK1)	AKI88007.1	0.0145		2.21	2.13	Apoptosis; platinum drug resistance; tight junction
NF-kappa B inhibitor alpha (NF_k_Bα)	AET34918.1	0.0161	0.46	0.28	0.43	Apoptosis; Pathways in cancer; shigellosis
TGF-beta-activated kinase 1 (TGF)	AKV88638.1	0.0338	2.31	2.17		Apoptosis; NF-kappa B signaling pathway; Shigellosis; MAPK signaling pathway
Alcohol dehydrogenase class-P (ADP)	XP_019577393.1	0.0293		2.43	2.16	Glycolysis/Gluconeogenesis; fatty acid degradation; tyrosine metabolism
Palmitoyl-protein thioesterase 1 (PPT1)	XP_015686279.1	0.0082		0.27	0.31	Glycolysis/Gluconeogenesis; shigellosis
Hexokinase	ABO21409.1	2.58E-06	3.23	3.32		Glycolysis/Gluconeogenesis; fatty acid degradation; drug metabolism—cytochrome P450
Alcohol dehydrogenase class II (Alcohol)	CCQ25768.1	0.0275		0.45	0.48	Insulin signaling pathway; pyruvate metabolism; AMPK signaling pathway
Acetyl-CoA carboxylase (Acetyl)	ALK82309.1	8.70E-05	3.23	3.13		Lysosome; fatty acid elongation

### qPCR Verification of Important Differentially Expressed Genes

**FIGURE 4:**
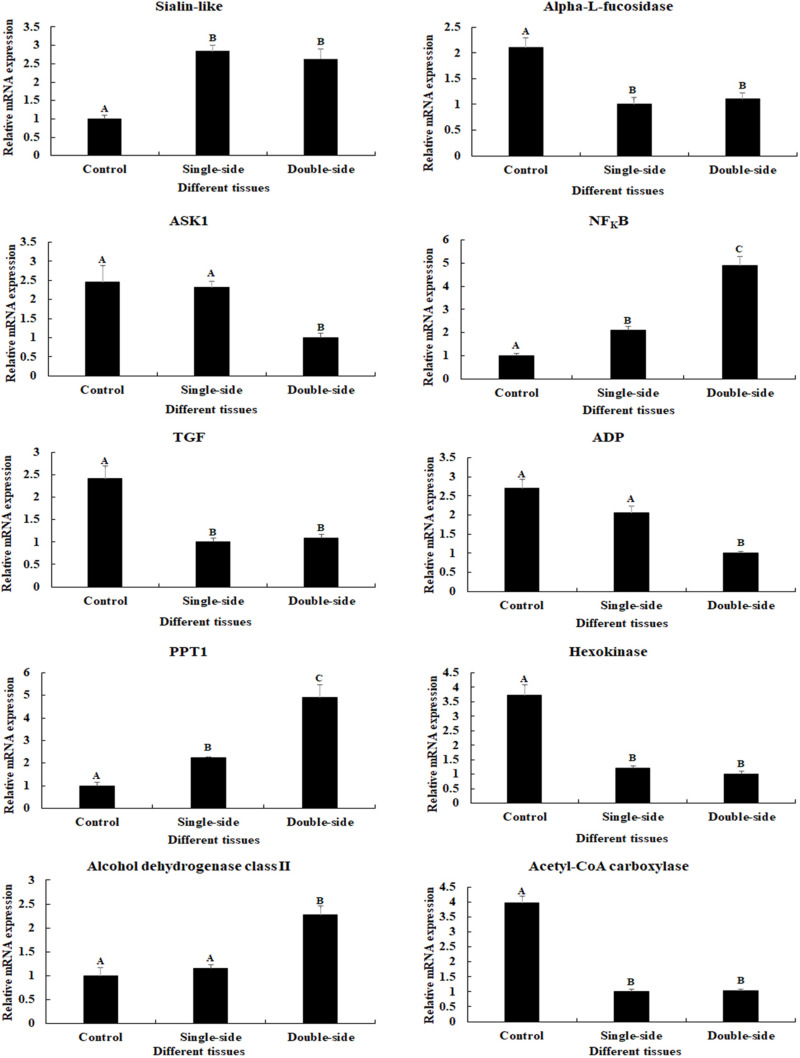
Verification of the expressions of 10 differentially expressed genes (DEGs) by qPCR. The amounts of DEG expression were normalized to the EIF transcript level. Data are shown as mean ± SD (standard deviation) of tissues in three separate individuals. Capital letters indicate expression difference. Control indicates normal prawns; single-side indicates the single-side ablation of eyestalk; double-side indicates the double-side ablation of eyestalk.

The expressions of 10 important DEGs were verified by qPCR, which showed the same expression pattern with that of RNA-Seq (Figure 4). The expressions of NF_K_Bα and PPT1 were gradually increased from the control group to double-side ablation and showed a significant difference between each group (*p* < 0.05). The lowest expression of sialin-like was observed in the control group and showed a significant difference with that of single-side ablation and double-side ablation (*p* < 0.05), while the highest expressions of alpha-L-fucosidase, TGF, HXK, and ACC were observed in the control group and showed a significant difference with that of single-side ablation and double-side ablation (*p* < 0.05). The highest expression of Alcohol dehydrogenase class II was observed in the double-side ablation and showed a significant difference with that of the control group and single-side ablation (*p* < 0.05), which the lowest expressions of ASK1 and ADP were observed in double-side ablation and showed a significant difference with that of the control group and single-side ablation (*p* < 0.05).

### qPCR Analysis of Mn-NF_k_Bα

**FIGURE 5:**
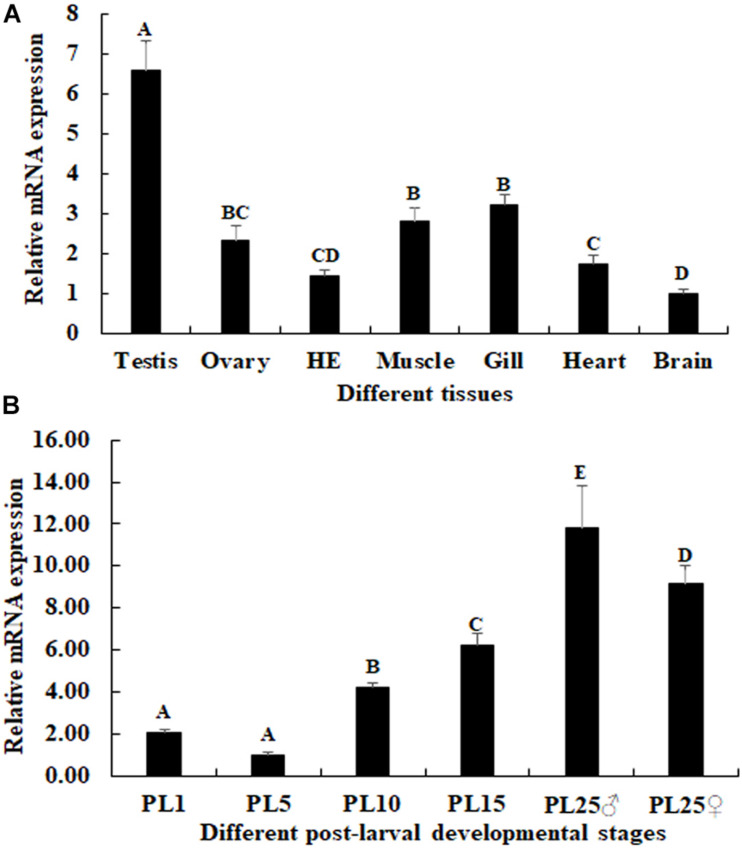
Expression characterization of Mn-NF_k_Bα in different tissues and post-larval developmental stages. The amount of Mn-NF_k_Bα mRNA was normalized to the EIF transcript level. Data are shown as mean ± SD (standard deviation) of tissues from three separate individuals. Capital letters indicate expression difference between different samples. **(A)** The expression characterization of Mn-NF_k_Bα in different tissues. **(B)** The expression characterization of Mn-NF_k_Bα in different post-larval developmental stages.

The physiology functions of a gene can be reflected by the qPCR analysis. According to the qPCR analysis in different tissues, the highest expression of Mn-NF_k_Bα was observed in the testis, which was significantly higher than the other tested tissues and showed a significant difference (*p* < 0.05; Figure 5A). The lowest expression was observed in the brain. The expression in the testis was 6.58-fold higher than that of the brain.

The mRNA expression of Mn-NF_k_Bα was also measured during the post-larval developmental stages. The PCR analysis revealed that the expression of Mn-NF_k_Bα was gradually increased in time with specimen development (Figure 5B). The gonad can be distinguished for the first time by the naked eye at PL25. The expression of Mn-NF_k_Bα was higher at both PL25♂ and PL25♀ and showed a significant difference with that of other developmental stages (*p* < 0.05). However, the expression at PL25♂ was higher than that of PL25♀ (*p* < 0.05). The lowest expression was observed in PL5, and the expressions in PL25♂ and PL25♀ were 11.83- and 9.15-fold higher than those of PL5, respectively.

### *In situ* Hybridization of Mn-NF_k_Bα

**FIGURE 6:**
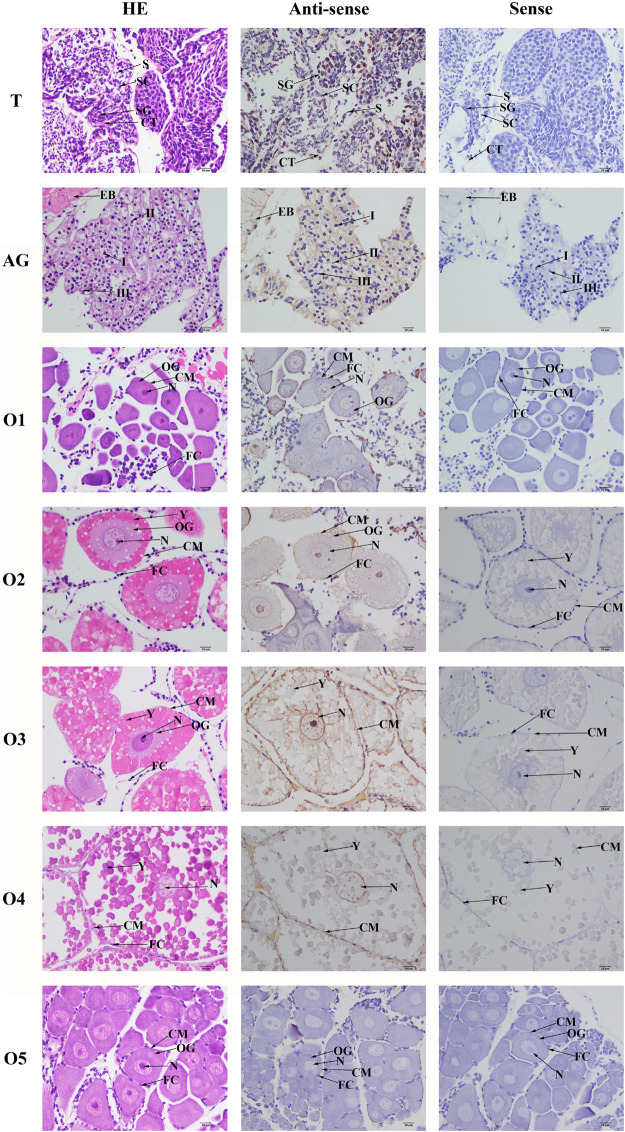
*In situ* hybridization analysis of Mn-NF_k_Bα gene in the testis and androgenic gland from reproductive season, and different reproductive cycle of ovary of *M. nipponense*. SG, spermatogonia; SC, spermatocytes; S, sperms; CT, collected tissue; I, Stage I of androgenic gland cell; II, Stage II of androgenic gland cell; III, Stage III of androgenic gland cell; EB, ejaculatory bulb; OG, oogonium; OC, oocyte; CM, cytoplasmic membrane; N, nucleus; Y, yolk granule; and FC, follicle membrane. Scale bars = 20 μm.

The cell type was labeled, based on the previous study (Figure 6). According to the *in situ* hybridization analysis, signals of Mn-NF_k_Bα were observed in spermatogonia and spermatocytes, whereas no signal was observed in sperms. Strong mRNA signals in the androgenic gland were only observed in the ejaculatory bulb surrounding the androgenic gland cells, while no signals were directly found in all stages of androgenic gland cells (Figure 6). Clear signals were rarely observed in O I and O V, while signals were observed in the nucleus, yolk granule, yolk granule, and cytoplasmic membrane in O II, O III, and O IV.

### The RNA Interference Analysis of Mn-NF_k_Bα

**FIGURE 7:**
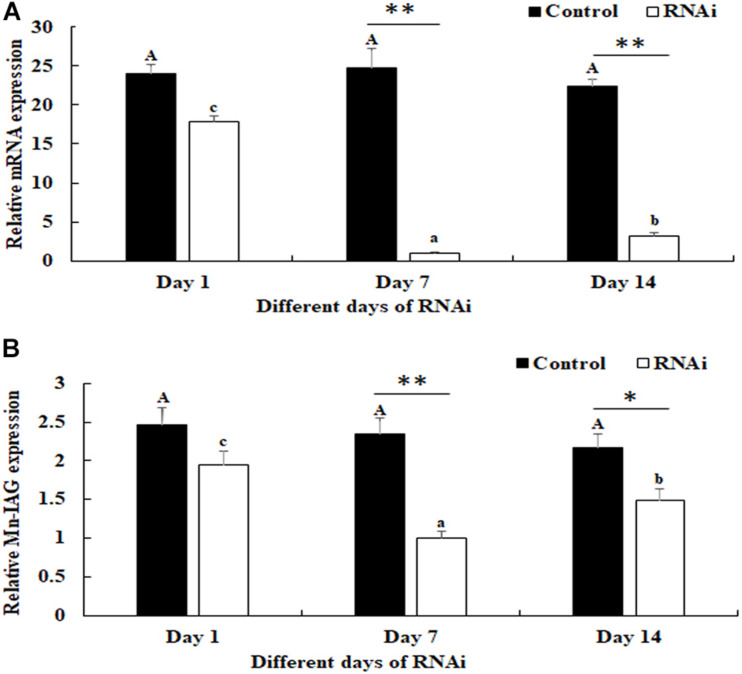
Expression characterization of Mn-NF_k_Bα and Mn-IAG at different days after Mn-NF_k_Bα dsRNA injection. The amount of Mn-NF_k_Bα and Mn-IAG mRNA was normalized to the EIF transcript level. Data are shown as mean ± SD (standard deviation) of tissues from three separate individuals. Capital letters indicate expression difference between different days after green fluorescent protein (GFP) injection in the control group. Lowercase letters indicate expression difference between different days after Mn-NF_k_Bα dsRNA injection in the RNA interference (RNAi) group. * (*p* < 0.05) and ** (*p* < 0.01) indicate significant expression difference between the RNAi group and control group at the sample day. **(A)** Expression characterization of Mn-NF_k_Bα at different days after Mn-NF_k_Bα dsRNA injection. **(B)** Expression characterization of Mn-IAG at different days after Mn-NF_k_Bα dsRNA injection.

The potential functions of Mn-NF_k_Bα on male sexual development in *M*. *nipponense* were analyzed by using RNAi. The expression levels of Mn-NF_k_Bα were measured in the testis after the treatment of Mn-NF_k_Bα dsRNA. According to the qPCR analysis, the expression of Mn-NF_k_Bα remained stable in the control group after the injection of GFP and showed no significant difference (*p* > 0.05). However, the expression of Mn-NF_k_Bα significantly decreased at days 7 and 14 after the injection of Mn-NF_k_Bα dsRNA. The decrease reached 95 and 85% at days 7 and 14, respectively, compared with that in the control group (Figure 7A).

The expressions of Mn-IAG were also measured in the androgenic gland from the same prawns (Figure 7B). According to the qPCR analysis, the expression of Mn-IAG at day 1 in the control group was slightly higher than that of day 7 and day 14, while it generally remained stable. In the RNAi group, the expressions of Mn-IAG were significantly decreased at day 7 and day 14 after the injection of Mn-NF_k_Bα dsRNA. The expression decreased about 61 and 54% at days 7 and 14, respectively, compared with that in the control group.

### Histological Observations of the Testis After RNA Interference

**FIGURE 8:**
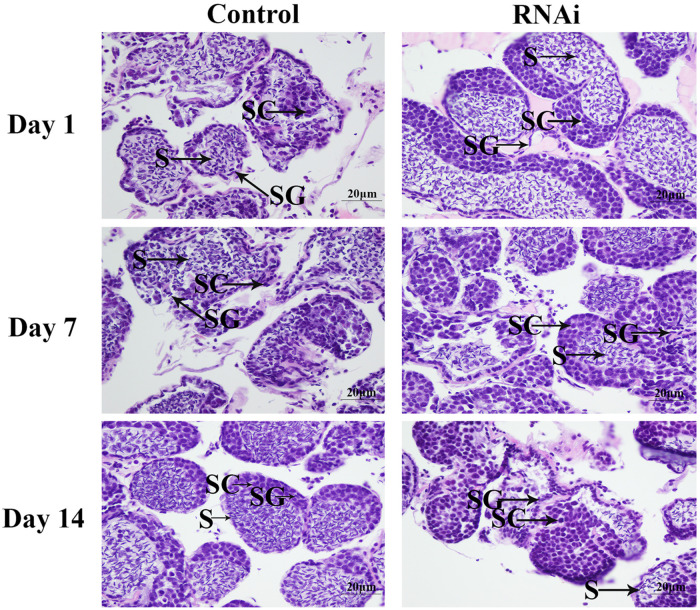
The morphological differences of the testis between the RNA interference (RNAi) and control groups. SG, spermatogonia; SC, spermatocytes; S, sperms; and CT, collected tissue. Scale bars = 20 μm.

According to the histological observations, the number of sperms was more than that of spermatogonia and spermatocytes in the control groups. Compared with that of the control group at day 7 and day 14, the number of sperms in the RNAi group was significantly decreased. In the RNAi group, the number of sperms was gradually decreased in time with Mn-NF_k_Bα dsRNA treatment, and sperms were rarely found at day 14 after Mn-NF_k_Bα dsRNA treatment (Figure 8).

## Discussion

The eyestalk of crustaceans secreted many neurosecretory structures and mediated the reproduction, molting, and metabolism of glucose in crustaceans (Jin et al., 2013b; Qiao et al., 2015, 2018). In this study, we aimed to analyze the regulatory effects on male sexual development through performing the transcriptome profiling analysis of the testis after eyestalk ablation. The histological observations of the testis after eyestalk ablation from *M. nipponense* indicated that the number of sperms in the DS prawns was significantly more than that of SS prawns and CG prawns, and spermatogonia were rarely observed in the DS prawns. This indicated that the hormones secreted by the eyestalk have negative regulatory effects on the testis development. This is the same as the results of a previous study that the hormones secreted by eyestalk inhibit the expression of IAG in *M. nipponense* (Li et al., 2015), and IAG promoted the male sexual characteristic development in many crustacean species (Ventura et al., 2009, 2011).

A total of 54,341 transcripts were generated in this study, providing valuable evidences on the studies of male sexual development. According to the GO and COG analyses, the genes related to the male sexual development were predicted to be mainly found in the functional groups of Cell, Cell part, Cellular process, and Binding in the GO assignment, and in the functional groups of General function prediction only, Signal transduction mechanisms, and Posttranslational modification, protein turnover, and chaperones in the COG classification, which were consistent with the previous studies (Jin et al., 2017, 2020). The number of DEGs between CG vs SS, SS vs DS, and CG vs DS was 1,039, 1,226, and 3,682, respectively, indicating that the ablation of double-side eyestalk has more regulatory effects on male sexual development than the single-side ablation in *M*. *nipponense*, which was consistent with histological observations of the testis after eyestalk ablation. KEGG analysis revealed that Lysosome, Apoptosis, Insulin signaling pathway, and Glycolysis/Gluconeogenesis were the main enriched metabolic pathways in all of these three comparisons, predicting that these mainly enriched metabolic pathways and vital DEGs from these metabolic pathways may play essential roles in male sexual development in *M*. *nipponense*. qPCR verification of these DEGs showed the same expression pattern with that of RNA-Seq, indicating the accuracy of the RNA-Seq.

Lysosomes are organelles playing essential roles in decomposing proteins, nucleic acids, polysaccharides, and other biological macromolecules. Lysosomes contain many hydrolases, functioning in the decomposition of the substances that enter into cells from the outside or the digestion of the local cytoplasm or organelles in the cells. The lysosomes will rupture, and the hydrolases will be released to digest the whole cells when cells are aged (Duve and Wattiaux, 1966; Luzio et al., 2007). Apoptosis refers to the programmed cell death, carefully controlled by genes to maintain the stability of internal environment. Apoptosis is an active process, which is different from cell necrosis. The process of apoptosis involves the activation, expression, and regulation of a series of genes, in order to better adapt to the living environment. Apoptosis plays important roles in the mechanism of physiology and pathology, in order to respond various stimuli, including ischemia, hypoxia, exposure to certain drugs and chemicals, immune reactions, infectious agents, high temperature, radiation, and various disease states (Thompson, 1995; Johnstone et al., 2002). According to the histological observations, the testis development was vigorous after eyestalk ablation. Thus, Lysosomes and Apoptosis were needed to digest the aged cells and to adapt to stress, in order to maintain the normal testis development. Alpha-L-fucosidase was a lysosomal enzyme, presented in all mammalian cells. Its activity has been proven to be deficient in the human autosomal recessive disease fucosidosis. It is also proposed as a marker of hepatocellular carcinoma (Fukushima et al., 1985). ACC is the enzyme playing essential roles in the synthesis of malonyl-coenzyme A (malonyl-CoA; Winder and Hardie, 1996). Malonyl-CoA is a key metabolite in the regulation of energy homeostasis. Malonyl-CoA was an inhibitor of fatty acid oxidation in skeletal muscle mitochondria, decreased in rat skeletal muscle during exercise or in response to electrical stimulation (Abu-Elheiga et al., 2001). ASK1 is one of the stress-responsive MAPK kinase kinase. ASK1 plays an important role in the response to reactive oxygen species, endoplasmic reticulum stress, and pro-inflammatory cytokines (Kawarazaki et al., 2014). It is involved in the pathogenesis of various diseases, including cancer, neurodegenerative diseases, infections, diabetes, and cardiovascular diseases (Liu et al., 2000). Nuclear factor kappa B (NF_k_B) plays essential roles in activating immune responses to exogenous stimuli and indigenous stimulation. NF_k_B proteins translocate into the nucleus to perform its functions during the stress condition (Thanos and Maniatis, 1995). NF_k_B expression was found in almost all cell types and tissues (Oeckinghaus and Ghosh, 2009). Furthermore, NF_k_B is involved in the mechanism of many processes, including immune and inflammatory responses, stress responses, and regulation of cell proliferation and apoptosis (Oeckinghaus and Ghosh, 2009).

Glycolysis/gluconeogenesis promotes the conversion of glucose (C_6_H_12_O_6_) into pyruvate (CH_3_COCOO- + H+), releasing free energy to form the high-energy molecule ATP and reduced nicotinamide adenine dinucleotide (Lubert, 1995). The main precursors of Glycolysis/gluconeogenesis include lactic acid, pyruvic acid, amino acid, and glycerol. In mammals, the process of Glycolysis/gluconeogenesis mainly occurred in the liver. The Glycolysis/gluconeogenesis ability of the kidney is only 10% of that of the liver in a normal condition. However, the ability of Glycolysis/gluconeogenesis in the kidney will significantly enhance at a starved condition for a long time. HXK proteins play important roles in catalyzing hexose phosphorylation and sugar sensing and signaling. Sucrose must be cleaved in hexoses (glucose and fructose) during the sucrose metabolism (Siemens et al., 2011). The hexoses are phosphorylated by HXKs or fructokinases. The phosphorylated hexoses are then involved in metabolic processes (David-Schwartz et al., 2013). PPT1 plays essential roles in catalyzing the hydrolysis of lipid thioesters on *S*-acylated proteins, which has a lysosomal localization and function in non-neuronal cells (Lu et al., 1996; Verkruyse and Hofmann, 1996; Lu et al., 2002). Individuals lacking functional PPT1 present with progressive psychomotor decline within the first year of life, followed by loss of vision and profound seizures, before entering a persistent vegetative state that invariably ends in premature death (Santavuori et al., 1973; Hofmann et al., 2002).

The functions of NF_k_Bα were further analyzed by qPCR, *in situ* hybridization, RNAi, and histological observations, because NF_k_Bα was the most up-regulated gene in the double-side eyestalk ablation prawns. The previous studies and histological observation revealed that the hormones secreted by eyestalk have negative effects on testis development (Sroyraya et al., 2010; Chung et al., 2011; Guo et al., 2019). The histological observations after the ablation of eyestalk in *M. nipponense* revealed that the testis development became vigorous after eyestalk ablation, and RNA-Seq analysis predicted that Apoptosis played essential roles in the maintaining the normal testis development after eyestalk ablation through digesting the aged cells. NF_k_Bα is enriched in the metabolic pathway of Apoptosis, which has essential DEGs, differentially expressed in all of these three comparisons. Thus, the significant up-regulation of NF_k_Bα expression after eyestalk ablation indicated that NF_k_Bα was predicted as a strong candidate gene for the mechanism of male sexual development in *M. nipponense*. The previous studies reported that the activation and repression of the NF_k_Bα expression were involved in the pathogenesis of inflammatory diseases, such as adult respiratory distress syndrome (ARDS) and breast cancers (Blackwell and Christman, 1997; Biswas et al., 2001). NF_k_Bα was proven to be involved in the immune system of *Macrobrachium rosenbergii*, because the expressions of NF_k_Bα were up-regulated after the feed of scutellaria polysaccharide and soybean antigen protein (Yang et al., 2019). However, to the best of our knowledge, no study has reported the potential functions of NF_k_Bα in the mechanism of male sexual development in any species. The qPCR analysis in different mature tissues revealed that the highest expression of Mn-NF_k_Bα was observed in the testis, which was significantly higher than the other tested tissues and showed a significant difference with other tested tissues, indicating that Mn-NF_k_Bα may have potential functions during the testis development in *M. nipponense*. qPCR was also used to measure the Mn-NF_k_Bα expression in post-larval developmental stages of *M. nipponense*. The results revealed that the Mn-NF_k_Bα expression was gradually increased with the specimen development, and PL25♂ showed higher expression than that of PL25♀. The sensitive period of gonad differentiation and development of *M. nipponense* has been proven to be from PL7 to PL22 (Jin et al., 2016). Thus, Mn-NF_k_Bα was predicted to play essential roles in male sexual development in *M. nipponense*, combined with the qPCR analysis in different mature tissues and post-larval developmental stages. *In situ* hybridization revealed that signals were observed in spermatogonia and spermatocytes, indicating that Mn-NF_k_Bα played essential roles in the testis development in *M. nipponense*. No signal was directly observed in the androgenic gland cells, while strong signals were observed in the ejaculatory bulb surrounding the androgenic gland cells, indicating that Mn-NF_k_Bα has potential functions in maintaining the normal functions and structures of androgenic gland in *M. nipponense* (Jin et al., 2018, 2019). In different ovarian developmental stages, no signal was observed in O I and O V, while signals were observed in the nucleus, yolk granule, yolk granule, and cytoplasmic membrane in O II, O III, and O IV, indicating that Mn-NF_k_Bα promotes yolk accumulation in *M. nipponense* (Li et al., 2018). RNAi analysis revealed that the ds-RNA of Mn-NF_k_Bα can efficiently knockdown the expression of Mn-NF_k_Bα in *M. nipponense*. In addition, the expression of Mn-IAG was also decreased with the decrease of Mn-NF_k_Bα, indicating that Mn-NF_k_Bα has a positive regulatory relationship with Mn-IAG. Thus, Mn-NF_k_Bα was involved in the male sexual development in *M. nipponense*, based on the importance of IAG in the male sexual development in crustacean species (Ventura et al., 2009, 2011, 2012). Histological observations after the treatment of Mn-NF_k_Bα dsRNA revealed that the number of sperms was decreased with the time of Mn-NF_k_Bα dsRNA treatment, indicating that Mn-NF_k_Bα has positive effects on testis development in *M. nipponense*.

In conclusion, histological observations revealed that eyestalk has negative effects on male sexual development in *M. nipponense*. A total of 1,039, 1,226, and 3,682 DEGs were identified between CG vs SS, SS vs DS, and CG vs DS, respectively, indicating that the ablation of double-side eyestalk has more regulatory roles on male sexual development in *M*. *nipponense*. Lysosome, Apoptosis, Glycolysis/Gluconeogenesis, and Insulin signaling pathway were the main enriched metabolic pathways in all of these three comparisons, and 10 important genes from these metabolic pathways were also selected. The functional analysis of NF_k_Bα by qPCR, RNAi, and histological observations revealed that NF_k_Bα has a positive regulatory effect on testis development in *M. nipponense*. This study identified the important functions of NF_k_Bα in male sexual development in *M. nipponense*, providing new insights for the construction of the technique to regulate the testis development. Crisper9 techniques will be further used to knock out the gene expression of NF_k_Bα in *M. nipponense* and to identify whether NF_k_Bα is also an important gene in the mechanism of sex determination in *M. nipponense*, resulting in the sea reversal.

## Data Availability Statement

The data presented in the study are deposited in the NCBI repository, accession numbers: SRX9832767–SRX9832775.

## Ethics Statement

The animal study was reviewed and approved by *Macrabrachium nipponense* the committee of Freshwater Fisheries Research Center and the Tai Lake Fishery Management Council. Written informed consent was obtained from the owners for the participation of their animals in this study.

## Author Contributions

ShJ designed and wrote the manuscript. HF supervised the study. YH performed the eyestalk ablation and transcriptome profiling analysis. YF and YG revised the manuscript. HQ performed the qPCR analysis. WZ performed the *in situ* hybridization analysis. YX performed the RNAi analysis. YW performed the histological observations. All authors contributed to the article and approved the submitted version.

## Conflict of Interest

The authors declare that the research was conducted in the absence of any commercial or financial relationships that could be construed as a potential conflict of interest.

## Footnotes

1 http://www.flyrnai.org/cgibin/RNAifind_primers.pl

## Supplementary Material

Supplementary Table 1 Summary of BLASTx results for unigenes of testis *M*. *nipponense* transcriptome.

## References

[B1] Abu-Elheiga L. Matzuk M. M. Abo-Hashema K. A. Wakil S. J. (2001). Continuous fatty acid oxidation and reduced fat storage in mice lacking acetyl-CoA carboxylase 2. *Science* 291 2613–2616. 10.1126/science.1056843 11283375

[B2] Almeida E. A. Petersen R. L. Andreatta E. R. Bainy A. C. (2004). Effects of captivity and eyestalk ablation on antioxidant status of shrimps (*Farfantepenaeus paulensis*). *Aquaculture* 238 523–528. 10.1016/j.aquaculture.2004.04.010

[B3] Ashburner M. Ball C. A. Blake J. A. Botstein D. Butler H. Cherry J. M. (2000). Gene ontology: tool for the unification of biology. *Nat. Genet.* 25 25–29. 10802651 10.1038/75556 PMC3037419

[B4] Benjamini Y. Drai D. Elmer G. Kafkafi N. Golani L. (2001). Controlling the false discovery rate in behavior genetics research. *Behav. Brain. Res.* 125 279–284. 10.1016/s0166-4328(01)00297-2 11682119

[B5] Biswas D. K. Dai S. C. Cruz A. Weiser B. Graner E. Pardee A. B. (2001). The nuclear factor kappa B (NF-κB): a potential therapeutic target for estrogen receptor negative breast cancers. *Proc. Natl. Acad. Sci. U.S.A.* 98 10386–10391. 10.1073/pnas.151257998 11517301 PMC56970

[B6] Blackwell T. S. Christman J. W. (1997). The role of nuclear factor-kappa B in cytokine gene regulation. *Am. J. Resp. Cell. Mol.* 17 3–9. 10.1165/ajrcmb.17.1.f132 9224203

[B7] Bolger A. M. Lohse M. Usadel B. (2014). Trimmomatic: a flexible trimmer for Illumina sequence data. *Bioinformatics* 30 2114–2120. 10.1093/bioinformatics/btu170 24695404 PMC4103590

[B8] Cai Y. Shokita S. (2006). Report on a collection of freshwater shrimps (Crustacea: Decapoda: Caridea) from the Philippines, with descriptions of four new species. *Raffles B. Zool.* 54 245–270.

[B9] Cao J. X. (2006). *The Molecular Mechanism in Male Reproductive Tract of the Prawn, Macrobrachium Rosenbergii.* Hangzhou: Zhejiang University.

[B10] Chung J. S. Manor R. Sagi A. (2011). Cloning of an insulin-like androgenic gland factor (IAG) from the blue crab, *Callinectes sapidus*: implications for eyestalk regulation of IAG expression. *Gen. Comp. Endocrinol.* 173 4–10. 10.1016/j.ygcen.2011.04.017 21596044

[B11] David-Schwartz R. Weintraub L. Vidavski R. Zemach H. Murakhovsky L. Swartzberg D. (2013). The SLFRK4 promoter is active only during late stages of pollen and anther development. *Plant Sci.* 199 61–70. 10.1016/j.plantsci.2012.09.016 23265319

[B12] Diarte-Plata G. Sainz-Hernández J. C. Aguinaga-Cruz J. A. Fierro-Coronado J. A. Polanco-Torres A. Puente-Palazuelos C. (2012). Eyestalk ablation procedures tominimize pain in the freshwater prawn *Macrobrachium Americanum*. *Appl. Anim. Behav. Sci.* 140 172–178. 10.1016/j.applanim.2012.06.002

[B13] Duve C. D. Wattiaux R. (1966). Function of lysosomes. *Annu. Rev. Physiol.* 28 435–492. 5322983 10.1146/annurev.ph.28.030166.002251

[B14] Fukushima H. Wet J. R. O’Brien J. S. (1985). Molecular cloning of a cDNA for human alpha-L-fucosidase. *Proc. Natl. Acad. Sci. U.S.A.* 82 1262–1265. 10.1073/pnas.82.4.1262 2983333 PMC397235

[B15] Grabherr M. G. Haas B. J. Yassour M. Levin J. Z. Thompson D. A. Amit I. (2011). Trinity: reconstructing a full-length transcriptome without a genome from RNA-Seq data. *Nat. Biotechnol.* 29 644–652. 21572440 10.1038/nbt.1883 PMC3571712

[B16] Guo Q. Li S. Lv X. (2019). Sex-biased CHHs and their putative receptor regulate the expression of IAG gene in the shrimp *Litopenaeus vannamei*. *Front. Physiol.* 10:1525. 10.3389/fphys.2019.01525 PMC6933007 31920723

[B17] Guo Z. H. (2007). *Study on Proliferation and Differentiation of Spermatogenic Cells From Macrobrachium Nipponense in Vitro.* Baoding: Hebei University.

[B18] Hofmann S. L. Atashband A. Cho S. K. Das A. K. Gupta P. Lu J. Y. (2002). Neuronal ceroid lipofuscinoses caused by defects in soluble lysosomal enzymes (CLN1 and CLN2). *Curr. Mol. Med.* 2 423–437. 10.2174/1566524023362294 12125808

[B19] Hopkins P. M. (2012). The eyes have it: a brief history of crustacean neuroendocrinology. *Gen. Comp. Endocrinol.* 175 357–366. 10.1016/j.ygcen.2011.12.002 22197211

[B20] Hu Y. N. Fu H. T. Qiao H. Sun S. M. Zhang W. Y. Jin S. B. (2018). Validation and evaluation of reference genes for Quantitative real-time PCR in *Macrobrachium nipponense*. *Int. J. Mol. Sci.* 19:2258. 10.3390/ijms19082258 30071669 PMC6121487

[B21] Jiang F. W. Fu H. T. Qiao H. Zhang W. Y. Jiang S. F. Xiong X. Y. (2014). The RNA interference regularity of transformer-2 gene of oriental river prawn *Macrobrachium nipponense*. *Chin. Agricult. Sci. Bul.* 30 32–37.

[B22] Jin S. B. Fu H. T. Jiang S. F. Xiong Y. W. Sun S. M. Qiao H. (2018). Molecular cloning, expression, and in situ hybridization analysis of forkhead box protein L2 during development in *Macrobrachium nipponense*. *J. World Aquacult. Soc.* 49 429–440. 10.1111/jwas.12510

[B23] Jin S. B. Fu H. T. Sun S. M. Jiang S. F. Xiong Y. W. Gong Y. S. (2017). Integrated analysis of microRNA and mRNA expression profiles at sex-differentiation sensitive period in oriental river prawn, *Macrobrachium nipponense*. *Sci. Rep.* 7:12011. 10.1038/s41598-017-10867-0 PMC5607309 28931848

[B24] Jin S. B. Fu H. T. Zhou Q. Sun S. M. Jiang S. F. Xiong Y. W. (2013a). Transcriptome analysis of androgenic gland for discovery of novel genes from the oriental river prawn, *Macrobrachium nipponense*, using Illumina Hiseq 2000. *PLoS One* 8:e76840. 10.1371/journal.pone.0076840 24204682 PMC3810145

[B25] Jin S. B. Hu Y. N. Fu H. T. Jiang S. F. Xiong Y. W. Qiao H. (2019). Potential functions of Gem-associated protein 2-like isoform X1 in the oriental river prawn *Macrobrachium nipponense*: Cloning, qPCR, *in situ* hybridization, and RNAi analysis. *Int. J. Mol. Sci.* 20:3995. 10.3390/ijms20163995 31426338 PMC6720513

[B26] Jin S. B. Wang N. Qiao H. Fu H. T. Wu Y. Gong Y. S. (2013b). Molecular cloning and expression of a full-length cDNA encoding crustacean hyperglycemic hormone (CHH) in oriental river pawn (*Macrobrachium nipponense*). *J. Fish. China.* 20 82–92. 10.3724/sp.j.1118.2013.00082

[B27] Jin S. B. Zhang Y. Guan H. H. Fu H. T. Jiang S. F. Xiong Y. W. (2016). Histological observation of gonadal development during post-larva in oriental river prawn, *Macrobrachium nipponense*. *Chin. J. Fish.* 29 11–16.

[B28] Jin S. Hu Y. N. Fu H. T. Jiang S. F. Xiong Y. W. Qiao H. (2020). Analysis of testis metabolome and transcriptome from the oriental river prawn (*Macrobrachium nipponense*) in response to different temperatures and illumination times. *Comp. Biochem. Phys. D* 34:100662. 10.1016/j.cbd.2020.100662 32114312

[B29] Johnstone R. W. Ruefli A. A. Lowe S. W. (2002). Apoptosis: a link between cancer genetics and chemotherapy. *Cell* 108 153–164. 11832206 10.1016/s0092-8674(02)00625-6

[B30] Kawarazaki Y. Ichijo H. Naguro I. (2014). Apoptosis signal-regulating kinase 1 as a therapeutic target. *Expert Opin. Ther. Tar.* 18 651–664. 10.1517/14728222.2014.896903 24660755

[B31] Li F. J. Bai H. K. Zhang W. Y. Fu H. T. Jiang F. W. Liang G. X. (2015). Cloning of genomic sequences of three crustacean hyperglycemic hormone superfamily genes and elucidation of their roles of regulating insulin-like androgenic gland hormone gene. *Gene* 561 68–75. 10.1016/j.gene.2015.02.012 25680292

[B32] Li F. Qiao H. Fu H. T. Sun S. M. Zhang W. Y. Jin S. B. (2018). Identification and characterization of opsin gene and its role in ovarian maturation in the oriental river prawn *Macrobrachium nipponense*. *Comp. Biochem. Physiol. B.* 218 1–12. 10.1016/j.cbpb.2017.12.016 29309912

[B33] Liu H. Nishitoh H. Ichijo H. Kyriakis J. M. (2000). Activation of apoptosis signal-regulating kinase 1 (ASK1) by tumor necrosis factor receptor-associated factor 2 requires prior dissociation of the ASK1 inhibitor thioredoxin. *Mol. Cell. Biol.* 20 2198–2208. 10.1128/mcb.20.6.2198-2208.2000 10688666 PMC110836

[B34] Livak K. J. Schmittgen T. D. (2001). Analysis of relative gene expression data using realtime quantitative PCR and the 2-ΔΔCT method. *Methods* 25 402–408. 10.1006/meth.2001.1262 11846609

[B35] Lu J. Y. Verkruyse L. A. Hofmann S. L. (1996). Lipid thioesters derived from acylated proteins accumulate in infantile neuronal ceroid lipofuscinosis. Correction of the defect in lymphoblasts by recombinant palmitoyl protein thioesterase. *Proc. Natl. Acad. Sci. U.S.A.* 93 10046–10050. 10.1073/pnas.93.19.10046 8816748 PMC38333

[B36] Lu J. Y. Verkruyse L. A. Hofmann S. L. (2002). The effects of lysosomotropic agents on normal and INCL cells provide further evidence for the lysosomal nature of palmitoyl-protein thioesterase function. *Biochim. Biophys. Acta* 1583 35–44. 10.1016/s1388-1981(02)00158-0 12069847

[B37] Lubert S. (ed.). (1995). “Glycolysis,” in *Biochemistry*, 4th Edn. (New York, NY: W.H. Freeman and Company), 483–508.

[B38] Luzio J. P. Pryor P. R. Bright N. A. (2007). Lysosomes: fusion and function. *Nat. Rev. Mol. Cell. Biol.* 8 622–632. 10.1038/nrm2217 17637737

[B39] Ma K. Y. Feng J. B. Lin J. Y. Li J. L. (2011). The complete mitochondrial genome of *Macrobrachium nipponense*. *Gene* 487 160–165. 10.1016/j.gene.2011.07.017 21827838

[B40] Ma X. K. Liu X. Z. Wen H. S. Xu Y. J. Zhang L. J. (2006). Histological observation on gonadal sex differentiation in *Cynoglossus semilaevis* Günther. *Mar. Fish. Res.* 27 55–61.

[B41] Minoru K. Michihiro A. Susumu G. Masahiro H. Mika H. Masumi I. (2008). KEGG for linking genomes to life and the environment. *Nucleic. Acids. Res.* 36 D480–D484. 18077471 10.1093/nar/gkm882 PMC2238879

[B42] Oeckinghaus A. Ghosh S. (2009). The NF-_*k*_B family of transcription factors and its regulation. *CSH Perspect. Biol.* 1:a000034. 10.1101/cshperspect.a000034 20066092 PMC2773619

[B43] Pamuru R. R. Rosen O. Manor R. Chung J. S. Zmora N. Glazer L. (2012). Stimulation of molt by RNA interference of the molt inhibiting hormone in the crayfish *Cherax quadricarinatus*. *Gen. Comp. Endocrinol.* 178 227–236. 10.1016/j.ygcen.2012.05.007 22664421

[B44] Qiao H. Fu H. T. Jin S. B. Wu Y. Jiang S. F. Gong Y. S. (2012). Constructing and random sequencing analysis of normalized cDNA library of testis tissue from oriental river prawn (*Macrobrachium nipponense*). *Comp*. *Biochem*. *Phys*. *D* 7 268–276. 10.1016/j.cbd.2012.04.003 22632994

[B45] Qiao H. Jiang F. W. Xiong Y. W. Jiang S. F. Fu H. T. Li F. (2018). Characterization, expression patterns of molt-inhibiting hormone gene of *Macrobrachium nipponense* and its roles in molting and growth. *PLoS One* 13:e0198861. 10.1371/journal.pone.0198861 29889902 PMC5995357

[B46] Qiao H. Xiong Y. Zhang W. Fu H. Jiang S. Sun S. M. (2015). Characterization, expression, and function analysis of gonad-inhibiting hormone in Oriental River prawn, *Macrobrachium nipponense* and its induced expression by temperature. *Comp. Biochem. Phys. A.* 185 1–8. 10.1016/j.cbpa.2015.03.005 25770669

[B47] Qiu G. F. Du N. S. Lai W. (1995). Studies on the male reproductive system of the freshwater prawn, *Macrobrachium nipponense*. *J*. *Shanghai Fish*. *Univ*. 4 107–111.

[B48] Revathi P. Vasanthi L. A. Jeyanthi S. Sankaralingam S. Ramasubburayan R. Prakash S. (2013). Impact of eyestalk ablation on the androgenic gland activity in the freshwater prawn *Macrobrachium rosenbergii* (De Man). *World* 5 373–381.

[B49] Sainz-Hernández J. C. Racotta I. S. Dumas S. Hernández-López J. (2008). Effect of unilateral and bilateral eyestalk ablation in Litopenaeus vannamei male and female on several metabolic and immunologic variables. *Aquaculture* 283 188–193. 10.1016/j.aquaculture.2008.07.002

[B50] Salma U. Uddowla M. H. Kim M. Kim J. M. Bo K. K. Baek H. J. (2012). Five hepatopancreatic and one epidermal chitinases froma pandalid shrimp (Pandalopsis japonica): cloning and effects of eyestalk ablation on gene expression. *Comp. Biochem. Phys. B* 161 197–207. 10.1016/j.cbpb.2011.11.005 22138334

[B51] Salman S. D. Page T. J. Naser M. D. Yasser A. G. (2006). The invasion of *Macrobrachium nipponense* (De Haan, 1849) (Caridea: Palaemonidae) into the southern Iraqi marshes. *Aquat. Invasions* 1 109–115. 10.3391/ai.2006.1.3.2

[B52] Santavuori P. Haltia M. Rapola J. Raitta C. (1973). Infantile type of so-called neuronal ceroid-lipofuscinosis: 1. A clinical study of 15 patients. *J. Neurol. Sci.* 18 257–267. 10.1016/0022-510x(73)90075-0 4698309

[B53] Santos E. A. Eduardo L. Nery M. Goncalves A. A. Keller R. (1997). Evidence for the involvement of the crustacean hyperglycemic hormone in the regulation of lipid metabolism. *Physiol. Biochem. Zool.* 70 415–420. 10.1086/515846 9237301

[B54] ShangGuan B. M. Liu Z. Z. Li S. Q. (1991). Histological studies on ovarian development in *Scylla serrata*. *J. Fish. China.* 15 96–103.

[B55] Shen H. Zhou X. Bai A. Ren X. Zhang Y. (2013). Ecdysone receptor gene from the freshwater prawn *Macrobrachium nipponense*: identification of different splice variants and sexually dimorphic expression, fluctuation of expression in the molt cycle and effect of eyestalk ablation. *Gen. Comp. Endocrinol.* 193 86–94. 10.1016/j.ygcen.2013.07.014 23899714

[B56] Siemens J. GonzáLez M. C. Wolf S. Hofmann C. Greiner S. Du Y. J. (2011). Extracellular invertase is involved in the regulation of clubroot disease in *Arabidopsis thaliana*. *Mol. Plant Pathol.* 12 247–262. 10.1111/j.1364-3703.2010.00667.x 21355997 PMC6640435

[B57] Sroyraya M. Chotwiwatthanakunab C. Stewartc M. J. Soonklangd N. Kornthong N. Phoungpetchara I. (2010). Bilateral eyestalk ablation of the blue swimmer crab, *Portunus pelagicus*, produces hypertrophy of the androgenic gland and an increase of cells producing insulin-like androgenic gland hormone. *Tissue Cell* 42 293–300. 10.1016/j.tice.2010.07.003 20817240

[B58] Tatusov R. L. Fedorova N. D. Jackson J. D. Jacobs A. R. Kiryutin B. Koonin E. V. (2003). The COG database: an updated version includes eukaryotes. *BMC Bioinformatics*. 4:41. 10.1186/1471-2105-4-41 PMC222959 12969510

[B59] Thanos D. Maniatis T. (1995). NF-KB: A lesson in family values. *Cell* 80 529–532. 10.1016/0092-8674(95)90506-5 7867060

[B60] Thompson C. (1995). Apoptosis in the pathogenesis and treatment of disease. *Science* 267 1456–1462. 10.1126/science.7878464 7878464

[B61] Tiu S. H. K. Chan S. M. (2007). The use of recombinant protein and RNA interference approaches to study the reproductive functions of a gonad-stimulating hormone from the shrimp *Metapenaeus ensis*. *FEBS J.* 274 4385–4395. 10.1111/j.1742-4658.2007.05968.x 17725713

[B62] Treerattrakool S. Chartthai C. Phromma-in N. Panyim S. Udomkit A. (2013). Silencing of gonad-inhibiting hormone gene expression in Penaeus monodon by feeding with GIH dsRNA-enriched Artemia. *Aquaculture* 404 116–121. 10.1016/j.aquaculture.2013.04.024

[B63] Treerattrakool S. Panyim S. Udomkit A. (2011). Induction of ovarian maturation and spawning in Penaeus monodon broodstock by double-stranded RNA. *Mar. Biotechnol.* 13 163–169. 10.1007/s10126-010-9276-0 20333425

[B64] Ventura T. Manor R. Aflalo E. D. Rosen O. Sagi A. (2012). Timing sexual differentiation: full functional sex reversal achieved through silencing of a single insulin-like gene in the prawn, *Macrobrachium rosenbergii*. *Biol. Reprod.* 86:90. 10.1095/biolreprod.111.097261 22133694

[B65] Ventura T. Manor R. Aflalo E. D. Weil S. Sagi A. (2009). Temporal silencing of an androgenic gland-specific insulin-like gene affecting phenotypical gender differences and spermatogenesis. *Endocrinology* 150 1278–1286. 10.1210/en.2008-0906 18988670

[B66] Ventura T. Manor R. Aflalo E. D. Weil S. Khalaila I. Rosen O. (2011). Expression of an androgenic gland-specific insulin-like peptide during the course of prawn sexual and morphotypic differentiation. *ISRN Endocrinol*. 2011:476283. 10.5402/2011/476283 PMC3262648 22363879

[B67] Verkruyse L. A. Hofmann S. L. (1996). Lysosomal targeting of palmitoyl protein thioesterase. *J. Biol. Chem.* 271 15831–15836. 10.1074/jbc.271.26.15831 8663305

[B68] Wang Y. B. Jin S. B. Fu H. T. Qiao H. Sun S. M. Zhang W. Y. (2019). Identification and characterization of the DMRT11E gene in the oriental river prawn *Macrobrachium nipponense*. *Int. J. Mol. Sci.* 20 1734. 10.3390/ijms20071734 30965605 PMC6480115

[B69] Winder W. W. Hardie D. G. (1996). Inactivation of acetyl-CoA carboxylase and activation of AMP-activated protein kinase in muscle during exercise. *Am. J. Physiol.* 270(2 Pt 1) E299–E304. 8779952 10.1152/ajpendo.1996.270.2.E299

[B70] Yang J. Guo Z. Cai X. Hua X. Liu T. Kong C. (2019). Physiological, biochemical, and immune effects of dietary soybean antigen proteins in the giant river prawn (*Macrobrachium rosenbergii*). *J. Fish. Sci. China* 26:322. 10.3724/sp.j.1118.2019.18092

[B71] Yang W. X. Du N. S. Lai W. (1999). Functional 646 relationship between spermatogenic cells and Sertoli cells during spermatogenesis of freshwater shrimp, *Macrobrachium nipponense*. *Acta Zool*. *Sin*. 45 178–186.

[B72] Zhang Y. P. Qiao H. Zhang W. Y. Sun S. M. Jiang S. F. Gong Y. S. (2013a). Molecular cloning and expression analysis two sex-lethal homolog genes during development in Oriental river prawn, *Macrobrachium nipponense*. *Genet*. *Mol*. *Res*. 12 4698–4711. 10.4238/2013.october.18.8 24222246

[B73] Zhang Y. P. Fu H. T. Qiao H. Jin S. B. Gong Y. S. Jiang S. F. (2013b). cDNA cloning, characterization and expression analysis of a transformer-2 gene in the oriental river prawn, *Macrobrachium nipponense*. *J. World Aquacult. Soc.* 44 338–349.

[B74] Zhang Y. P. Jiang S. F. Xiong Y. W. Sun S. M. Qiao H. Jin S. B. (2013c). Molecular cloning and expression analysis of extra sex combs gene during development in *Macrobrachium nipponense*. *Turk*. *J*. *Fish*. *Aquat*. *Sc*. 13 331–340.

